# Robotic versus laparoscopic one anastomosis gastric bypass (OAGB): a propensity score-matched comparative study of perioperative outcomes in 200 patients

**DOI:** 10.1007/s00464-026-12903-5

**Published:** 2026-05-26

**Authors:** Mario Musella, Antonio Franzese, Vincenzo Schiavone, Pasquale Avella, Alessandra D’Ambrosio, Lucrezia Borrelli, Carolina Bartolini, Gerardo D’Amato

**Affiliations:** Advanced Biomedical Sciences Department, Federico II University, Via Sergio Pansini, 5, 80131 Naples, Italy

**Keywords:** Metabolic bariatric surgery (MBS), Robotic bariatric surgery, Laparoscopic bariatric surgery, One anastomosis gastric bypass, Perioperative outcomes

## Abstract

**Background:**

Severe obesity poses a substantial health burden, often necessitating surgical intervention. One anastomosis gastric bypass (OAGB) has emerged as a reliable bariatric procedure. In recent years, robotic-assisted surgery has gained traction due to its potential advantages over conventional laparoscopic techniques. However, comparative data on the outcomes of robotic versus laparoscopic OAGB remain limited.

**Objectives:**

This pilot study aims to evaluate the surgical outcomes of robotic versus laparoscopic OAGB, focusing on safety, feasibility, and postoperative recovery. The primary endpoints include operative duration, intraoperative and postoperative complications, postoperative pain, and length of hospital stay.

**Materials and methods:**

A retrospective analysis was conducted on 241 patients who underwent primary OAGB at the Bariatric and Endocrine-Metabolic Surgery Unit, University of Naples "Federico II," between January 2023 and December 2025. After propensity score matching, 100 patients underwent robotic OAGB utilizing the Da Vinci Xi® system and 100 laparoscopic OAGB.

**Results:**

Baseline demographic characteristics were comparable between groups. The robotic-assisted procedure exhibited a longer mean operative time (112.90 ± 10.70 min vs. 107.70 ± 12.48 min; *p* = 0.018). No intraoperative complications or conversions to laparoscopy or open surgery were recorded. Robotic group reported lower postoperative pain scores on postoperative day 1 (*p* < 0.0001). No significant differences were observed regarding mean hospital stay, postoperative nausea, vomiting, or overall complication rates.

**Conclusions:**

These preliminary findings suggest that r-OAGB is an efficient and safe alternative to laparoscopic approach. However, larger prospective studies are warranted to validate these results and assess long-term outcomes and cost-effectiveness.

**Graphical abstract:**

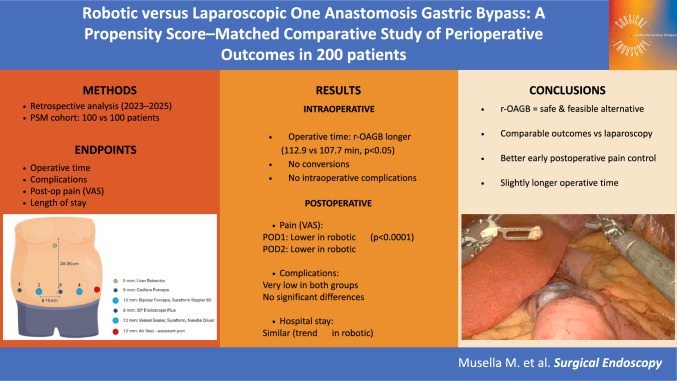

**Supplementary Information:**

The online version contains supplementary material available at 10.1007/s00464-026-12903-5.

Nowadays, metabolic and bariatric surgery (MBS) represent the gold standard for patients suffering from morbid obesity [1–3]. According to the American Society for Metabolic and Bariatric Surgery (ASMBS) and the International Federation for the Surgery of Obesity and Metabolic Disorders (IFSO), patients are suitable for surgical approaches in case of body mass index (BMI) > 35 kg/m^2^ or > 30 kg/m^2^ in the presence of comorbidities [4].

One anastomosis gastric bypass (OAGB) represents the third-most performed and popular procedure due to its effectiveness in terms of weight loss, gaining recognition and feasibility [5–7].

Nowadays, laparoscopy represents the conventional technique to perform laparoscopic one anastomosis gastric bypass (l-OAGB) [8]. Although the da Vinci robot has been increasingly used for robotic one anastomosis gastric bypass (r-OAGB), its use remains controversial [8].

The purpose of our comparison was to provide a comprehensive analysis of the efficacy and safety of the two surgical approaches, contributing valuable insights to the ongoing evolution of MBS practices. In addition, we seek to elucidate the potential benefits and drawbacks of r-OAGB and l-OAGB, thereby informing future clinical decisions and enhancing patient care.

## Materials and methods

### Study design and population

**Fig. 1:**
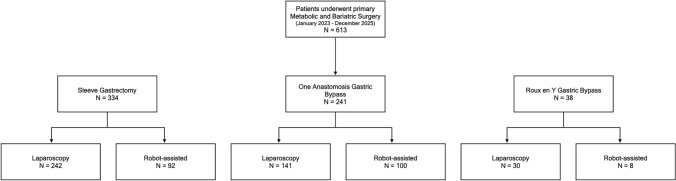
Distribution of primary metabolic bariatric surgery procedures performed from January 2023 to December 2025

We retrospectively reviewed a prospectively collected database of patients undergoing r-OAGB and l-OAGB at the Bariatric and Endocrine-Metabolic Surgery Unit of the University of Naples “Federico II” (Naples, Italy) from January 2023 to December 2025. During this period, 613 patients underwent primary metabolic bariatric surgery (MBS) at our unit: 100 (16.31%) patients experienced primary r-OAGB, while 141 (23%) primary l-OAGB (Fig. 1).

We selected, after propensity score matching (PSM), 100 consecutive r-OAGB and 100 l-OAGB in obese patients. All patients signed an informed consent allowing the anonymous scientific use of clinical data and images. The study was carried out according to the Declaration of Helsinki guidelines. Demographic, clinical, surgical, and pathological data were prospectively collected and retrospectively reviewed according to the Strengthening the Reporting of Observational Studies in Epidemiology (STROBE) statement [9].

Patients ≥ 18 and < 70 years old were included in our study. According to the Guideline of The Italian Society of Bariatric Surgery and Metabolic Diseases, patients with BMI ≥ 40 kg/m^2^ without comorbidities or ≥ 35 kg/m^2^ with comorbidities were candidates for surgery. All procedures were performed in elective surgery priority.

All patients included in this analysis underwent primary OAGB. Patients with a history of previous bariatric or upper gastrointestinal surgical procedures (i.e., revisional cases) were excluded from the study.

Furthermore, we excluded the first 21 r-OAGB performed during our Institution Learning Curve in Robotic Bariatric Surgery Program in 2022. All procedures were performed by a single expert laparoscopic surgeon.

### Endpoints

The primary outcome of interest was to compare the technical differences between r-OAGB and l-OAGB, performing a step-by-step analysis of both minimally invasive approaches. Operative time was defined as “skin-to-skin” minutes, registered by a dedicated team in the operating room, including robotic docking time.

Secondary outcomes involved pre-, intra-, and postoperative days (POD) variables analysis to identify predictive factors linked to major complications. Furthermore, we collected pain control data to detect any differences between r-OAGB and l-OAGB.

### Data collection

The surgeons’ team compiled a Microsoft Excel Database, including demographic data (e.g., age, sex, BMI), clinical variables such as American Society of Anesthesiologist (ASA) score [10], comorbidities, operative time (minutes), conversion to open surgery and intraoperative complications, Post-Operative Nausea and Vomiting (PONV) and Visual Analogue Scale (VAS) value at POD1, POD2 and POD3, and postoperative outcomes until 30-day after surgery.

Patient comorbidities were systematically stratified according to the Charlson Comorbidity Index (CCI) [11].

Postoperative morbidities were classified according to the Clavien-Dindo (CD) classification [12]. Severe surgical complications were defined as CD grade ≥ 3. Clinically, radiologically, or endoscopically anastomosis defect was categorized as anastomotic leaks.

### Preoperative management

Under the supervision of the Multidisciplinary Team (MDT), all patients performed a 3-week very low-carbohydrate ketogenic diet (VLCKD) program and psychological evaluation before surgery. Furthermore, an esophagogastroduodenoscopy (EGDS) was required during preoperative management.

In selected cases, spirometry, echocardiogram, and perioperative Continuous Positive Airway Pressure (CPAP) were performed. Patients were defined as “fit-to-surgery” when at least 10% of body weight was lost.

### Post-operative workup and follow-up

A dedicated Enhanced Recovery After Surgery (ERAS) program was used to achieve a rapid recovery of patients’ conditions [13].

A water-only diet was ongoing on POD2, while a liquid diet on POD3. Our institutional protocol targets discharge at POD3 in uncomplicated cases.

After two weeks, patients were advised to start with a semi-solid diet and gradually transition to a regular diet over the next 2 to 4 weeks. Multivitamin supplementation is administered for at least 1 year after surgery. In the absence of clinical signs of a leak, stenosis, or other complications, we scheduled for discharge at POD3. For the purpose of this study, each patient underwent a standardized interview conducted by the same attending physician on postoperative days 0, 1, and 2. During these interviews, the incidence and severity of postoperative nausea and vomiting (PONV) were meticulously assessed, alongside the patient’s perceived pain intensity, which was quantified using the Visual Analog Scale (VAS) score.

Surgical site infections (SSI) were monitored in both inpatient and outpatient settings to identify potential complications [14].

### Statistical analysis and propensity score matching

The quantitative data are reported as mean ± standard deviation (SD) and median (range). Normally distributed quantitative data were analyzed with a t-test. The qualitative data are presented as the number of patients (percentage) and a comparison was conducted using Fisher’s exact test. All tests were two-sided with a significance level of 5%. Statistical significance was defined as a *p*-value < 0.05.

The analyses were performed using IBM SPSS, version 23 (IBM Co., Armonk, NY, USA).

PSM was performed to overcome the bias due to patients’ selection and characteristics. We obtained the PSM using a logistic regression model that included: age, sex, ASA classification, preoperative BMI and CCI. After estimation of PSM, a regular 1:1 Nearest-Neighbor matching was carried out. The PSM was performed using XLSTAT statistical and data analysis solution (Lumivero 2023, Paris, France).

A power analysis was performed to determine the minimum sample size required to detect clinically meaningful differences between r-OAGB and l-OAGB. With a significance criterion of *α* = 0.05 and power = 0.80, the minimum sample size required is *N* = 88 [15]. Thus, the obtained sample size of *N* = 100 is deemed to be adequate to test the study hypothesis [16].

## Intraoperative technical notes

### l-OAGB

Patients were placed in 20° reverse Trendelenburg position with legs apart. Pneumoperitoneum was established using a Veress needle at Palmer’s point, with pressure set at 14–16 mmHg. A six-port (5 × 10 mm, 1 × 5 mm) approach was used. The gastric pouch was fashioned along a 36-Fr starting just below the crow’s foot. No reinforcement was routinely applied on the staple line. The biliopancreatic limb (BPL) was tailored on the patient’s BMI. The gastrojejunostomy was performed using a 45–mm linear stapler and enterotomies were closed by an anterior, double-layer, self- locking, running absorbable suture. A methylene blue test is performed. A fibrin glue was applied on the anastomosis and on the staple line. An abdominal drain was routinely placed behind the anastomosis [17].

### r-OAGB

The procedure was performed using the Da Vinci Xi® system. Patients were placed in 20° reverse Trendelenburg position with legs apart. The surgeon operated from the console, with an assistant positioned between the patient’s legs and a scrub nurse completing the surgical team. Pneumoperitoneum was established using a Veress needle at Palmer’s point, with pressure set at 14–16 mmHg. Trocar placement included one 8–mm optical trocar positioned approximately twenty-five centimeters from the xiphoid process and three additional robotic trocars along a perpendicular line to the xipho-umbilical axis. A 5-mm trocar was placed in the epigastric region for liver retractor. A 10-mm assistant trocar was placed on the left side. Docking of robotic arms followed, and robotic graspers, advanced bipolar energy instruments, and staplers were used. The procedure involved gastric pouch creation, anastomosis with a jejunal loop 180–220 cm from the Treitz ligament (tailored on the patient’s BMI). A 45 mm gastrojejunostomy was performed using a linear stapler and enterotomies were closed by an anterior, double-layer, self- locking, running absorbable suture. A methylene blue test is performed. A fibrin glue was applied on the anastomosis and on the staple line. An abdominal drain was routinely placed behind the anastomosis (Supplementary Video).

## Results

A total of 241 patients undergoing primary were included before matching, of whom 100 (41.49%) underwent r-OAGB and 141 (58.51%) l-OAGB. After matching, 200 patients were analyzed (100 r-OAGB and 100 l-OAGB). Demographic and clinical characteristics are summarized in Table 1.

**Table 1:** Demographic and clinical perioperative data of patients underwent OAGB

	Before matching			After matching		
	r-OAGB	l-OAGB	*p *value	r-OAGB	l-OAGB	*p* value
Number of patients, (%)	100 (41.5)	141 (58.5)	–	100 (50)	100 (50)	–
Age, years; mean ± SD	42.70 ± 9.40	42.60 ± 7.23	*0.9257*	42.70 ± 9.40	42.0 ± 11.30	*0.6344*
Gender (F/M); n.ro (%)	80/20 (80/20)	102/39 (72.4/27.6)	*0.2237*	80/20 (80/20)	78/22 (80/20)	*0.9999*
CCI; mean ± SD	2.71 ± 0.45	2.89 ± 0.32	***0.0003***	2.71 ± 0.45	2.53 ± 0.50	***0.0081***
ASA score; n.ro (%)						
I	20 (20)	31 (22)	*0.7510*	20 (20)	16 (16)	*0.5813*
II	30 (30)	45 (32)	*0.7792*	30 (30)	28 (28)	*0.8763*
III	50 (50)	65 (44)	*0.6013*	50 (50)	56 (56)	*0.4788*
IV	0 (0)	0 (0)	*0.9999*	0 (0)	0 (0)	*0.9999*
BMI, kg/m^2^; mean (± SD)	46.70 ± 5.00	48.10 ± 6.12	*0.0607*	46.70 ± 5.00	47.80 ± 4.50	*0.1036*
Docking time, min; mean ± SD	12.06 ± 3.53	–	–	12.06 ± 3.53	–	–
Operative time, min; mean ± SD	112.90 ± 10.70	103.20 ± 21.13	** < *0.0001***	112.90 ± 10.70	107.70 ± 12.48	***0.0018***
Conversion to laparoscopy/open; n.ro (%)	0 (0)	0 (0)	*0.9999*	0 (0)	0 (0)	*0.9999*
PONV; mean ± SD						
POD 0	1.81 ± 0.98	1.83 ± 1.02	*0.8790*	1.81 ± 0.98	1.87 ± 0.72	*0.6223*
POD 1	0.62 ± 0.62	0.58 ± 0.67	*0.6382*	0.62 ± 0.62	0.75 ± 0.58	*0.1273*
POD 2	0.27 ± 0.50	0.31 ± 0.43	*0.5069*	0.27 ± 0.50	0.35 ± 0.45	*0.3351*
VAS; mean ± SD						
POD 0	6.67 ± 1.80	6.54 ± 1.64	*0.5610*	6.67 ± 1.80	6.76 ± 1.36	*0.6904*
POD 1	3.75 ± 1.65	4.76 ± 1.32	** < *0.0001***	3.75 ± 1.65	5.12 ± 1.31	** < *0.0001***
POD 2	1.25 ± 1.18	1.27 ± 0.97	*0.8856*	1.25 ± 1.18	1.69 ± 1.35	***0.0150***
Complications, n.ro (%)						
Abdominal wall bleeding	1 (1)	0 (0)	*0.4149*	1 (1)	0 (0)	*0.9999*
SSI	0 (0)	1 (0.7)	*0.9999*	0 (0)	1 (1)	*0.9999*
Length of stay, days; mean ± SD	4.30 ± 3.50	5.10 ± 3.42	*0.0777*	4.30 ± 3.50	5.60 ± 7.60	*0.1219*

Italics indicate the *p*-values. Bold indicates statistically significant values

Before matching, mean age and sex distribution were comparable between r-OAGB and l-OAGB (42.70 ± 9.40 *vs.* 42.60 ± 7.23 years, *p* = 0.9257; females 80% vs. 72%, *p* = 0.2237). In contrast, patients in the l-OAGB group presented with a significantly higher CCI (2.89 ± 0.32 *vs.* 2.71 ± 0.45, *p* = 0.0003) and a higher BMI (48.10 ± 6.12 vs. 46.70 ± 5.00 kg/m^2^, *p* = 0.0607). ASA distribution did not differ significantly between groups. After matching, baseline characteristics were well balanced: age, sex, ASA class and BMI were similar between r-OAGB and l-OAGB. However, the small residual difference in CCI reached statistical significance (2.71 ± 0.45 vs. 2.53 ± 0.50, *p* = 0.0081).

Intraoperative outcomes in the unmatched cohort showed a significantly longer operative time for r-OAGB compared with l-OAGB (112.90 ± 10.70 *vs.* 103.20 ± 21.13 min, *p* < 0.0001), with a mean docking time for the robotic platform of 12.06 ± 3.53 min. After matching, operative time remained slightly longer in the r-OAGB group (112.90 ± 10.70 *vs.* 107.70 ± 12.48 min, *p* = 0.0018). No conversions to conventional laparoscopy or open surgery occurred in either group, both before and after matching.

Postoperative complications were rare in both cohorts. Before matching, one case (1%) of abdominal wall bleeding occurred in the r-OAGB group and one surgical site infection (0.7%) in the l-OAGB group, with no statistically significant differences. Superimposable data are collected after matching.

PONV scores were low and comparable between groups at all postoperative time points (Table 1). Regarding postoperative pain, VAS scores did not differ significantly between groups at POD0 (6.67 ± 1.80 vs. 6.76 ± 1.36; *p* = 0.6904). However, the r-OAGB group demonstrated significantly lower VAS scores at POD1 (3.75 ± 1.65 vs. 5.12 ± 1.31; *p* < 0.0001) and POD2 (1.25 ± 1.18 vs. 1.69 ± 1.35; *p* = 0.0150), suggesting a more favorable early postoperative pain profile in the robotic group (Table 1).

Mean length of hospital stay was comparable between groups both before (4.30 ± 3.50 vs. 5.10 ± 3.42*p* = 0.0777) and after matching (4.30 ± 3.50 vs. 5.60 ± 7.60 *p* = 0.1219).

No anastomotic leaks, reoperations, or 30-day readmissions were recorded in either group. Biliary reflux was not systematically assessed at this early timepoint and is acknowledged as a limitation of the present study.

## Discussion

OAGB is a well-established procedure for treating obesity, providing significant weight loss and remission of obesity-related comorbidities. Both l- and r-OAGB approaches have been employed, each offering distinct advantages and challenges. In Italy, the robotic technique is gradually gaining ground, as reported by Fantola et al. [8]. Robotic OAGB ranks third among bariatric–metabolic procedures performed with the robotic approach up to 2024.

Robotic-assisted surgery provides unmatched dexterity and three-dimensional visualization, enhancing precision in tissue handling, dissection, and suturing [18]. While there is an inherent learning curve when adopting robotic platforms, our findings, without intraoperative complications or conversions, demonstrate the safety and feasibility of r-OAGB when performed by experienced surgeons. As robotic systems become more integrated into bariatric programs, we expect the learning curve to shorten and the technique to spread globally. Mastery of the robotic system requires substantial training and practice; however, once proficiency is achieved, it can enhance the surgeon’s capabilities, particularly in complex cases. In contrast, l-OAGB, having been established for longer, benefits from a larger pool of experienced practitioners and standardized protocols, which contribute to more consistent outcomes across various healthcare settings [19].

In our study, the decision to assign patients to r-OAGB or l-OAGB was primarily based on the availability of the robotic system on specific surgical days, rather than clinical criteria. This approach minimized selection bias and ensured a fair comparison between the two techniques, with comparable baseline demographic characteristics between the groups. Nevertheless, previous studies have highlighted the advantages of robotic technology in complex cases, such as patients with higher BMI, those undergoing revisional surgery, or those with difficult intra-abdominal anatomy. In these cases, enhanced dexterity, superior visualization, and greater precision are crucial [20].

Operative outcomes in our cohort showed that although the mean operative time for r-OAGB was approximately nine minutes longer than for l-OAGB, indicating that the robotic system’s docking significantly prolonged the procedure. Additionally, no intraoperative complications, mortality, or conversions to open surgery were observed in either group, supporting the safety and feasibility of both techniques when performed by experienced surgeons. Similarly, Zhang et al*.* [21]. conducted a meta-analysis of robotic versus laparoscopic bariatric procedures and found that robotic surgery was associated with longer operative times but comparable safety profiles, including low rates of intraoperative complications and conversions.

An important finding in our study was the significantly lower postoperative pain scores (VAS) in the robotic group on postoperative days 1 and 2, suggesting that the less traumatic tissue manipulation afforded by robotic systems may lead to improved patient comfort and faster recovery. Spurzem et al*.* [22] analyzed over 40,000 revisional bariatric cases and found that robotic surgery was associated with reduced morbidity and faster recovery compared to laparoscopy. This aligns with previous studies that link robotic surgery to reduced postoperative pain, lower analgesic requirements, and earlier discharge from the hospital [23, 24]. In our series, no statistically significant difference was observed in the mean length of hospital stay between patients undergoing robotic versus laparoscopic surgical approaches (*p* = 0.1219). However, a slight reduction in length of stay was noted in the robotic group. Although not significant in our cohort, this trend, if confirmed in larger series, could potentially translate into meaningful savings in overall hospitalization costs through reduced resource utilization, including nursing care, bed occupancy, and ancillary services.

Our OAGB was performed according to technical principles described by several experts, who recommend a gastrojejunostomy with a diameter ranging from 2.5 to 5 cm [25, 26]. Although anastomotic diameter has been discussed as a potential technical variable, the current literature does not demonstrate a clear, consensus-based association between a 45-mm anastomosis and marginal ulcer formation, and no uniform recommendation exists to systematically reduce the anastomotic diameter for ulcer prevention[27].

Cost considerations remain a significant point of debate in adopting robotic platforms. It should be noted that no formal cost-effectiveness analysis was performed in the present study; therefore, any economic considerations must be interpreted as speculative and informed by the existing literature rather than by data derived from our cohort. Based on current Italian Health System reimbursement rates reported in the literature (approximately 5,112.90€ per bariatric procedure), l-OAGB appears economically sustainable within the national reimbursement framework. Whether r-OAGB can achieve comparable economic sustainability remains uncertain, as direct procedural costs are potentially higher and remain non-standardized, varying according to region, hospital setting, and robotic platform utilized.

Clinical pathways have shown to reduce resource consumption and overall procedural costs [28, 29]. While robotic surgery is typically associated with higher direct procedural costs due to equipment acquisition, maintenance, and disposable instruments [30–32], the increased availability of minimally invasive surgery via robotic platforms may result in overall cost reduction compared to open procedures, especially when considering faster recovery, fewer postoperative complications, and reduced hospital resource utilization. Munshower et al*.* [33] performed a cost analysis of robotic general surgery and concluded that while robotic procedures are more expensive initially, they may offset these costs through reduced complications and shorter hospital stays. By decreasing postoperative morbidity and optimizing perioperative care, robotic systems could gradually amortize their initial investment, particularly in high-volume bariatric centers [23].

It is important to emphasize that the potential economic and clinical benefits of robotic surgery could be even more pronounced in high-risk or complex patients [34, 35]. Literature increasingly suggests that robotic platforms may improve outcomes in patients with extreme obesity (BMI > 50), those with prior abdominal surgeries, or those with significant visceral fat, where technical challenges are greater and surgical precision is crucial [20, 36, 37]. Marincola et al*.* [20] showed superior outcomes in super-obese patients undergoing robotic bariatric surgery, suggesting that the precision and dexterity of robotic systems are particularly beneficial in these technically demanding cases. While our cohort consisted of a general bariatric population, future studies should focus on high-risk groups to better define the optimal indications for r-OAGB and robotic MBS.

The field of robotic bariatric surgery is rapidly evolving, and recent studies on r-OAGB have increased significantly, reflecting growing interest in its application beyond primary bariatric interventions. For example, Zucchini et al.[38] described the first comparative data on 54 r-OAGB and 81 l-OAGB procedures, demonstrating that while laparoscopic surgery remains faster, the robotic platform ensures greater procedural consistency during the adoption phase. Reche et al*.* [39] described the first robotic reversal of omega-loop gastric bypass, demonstrating excellent surgical precision and supporting the versatility of robotic approaches in complex revisional surgery. Bhandari et al.[40] presented the first case series of 10 r-OAGB procedures performed via telesurgery, establishing the feasibility and clinical safety of long-distance tele-robotic bariatric surgery.

As robotic technology continues to advance, its integration into bariatric surgery will likely expand, bolstered by increasing evidence of its benefits [41]. Improvements in system accessibility, training programs, and operative efficiency will likely accelerate the adoption of robotic platforms in standard bariatric practice [8]. These results underscore the growing role of robotic platforms in enhancing surgical outcomes and optimizing resource allocation.

Our findings suggest that, with appropriate case selection and institutional investment, robotic surgery may absolutely match with traditional laparoscopy in the management of obesity, representing a potential paradigm shift in minimally invasive bariatric surgery. The choice between these techniques should ultimately be based on patient needs, surgeon expertise, and institutional resources.

## Strengths and limitations

Our study contributes to the growing body of evidence supporting robotic bariatric surgery, but several limitations must be acknowledged. This is a pilot study based on the preliminary experience of a single center. The single-center design and relatively small sample size may limit generalizability.

Although propensity score matching successfully balanced most baseline characteristics, a small but statistically significant residual difference in the CCI persisted after matching (2.71 ± 0.45 vs. 2.53 ± 0.50, *p* = 0.0081). Both values fall within CCI score range 2–3, which corresponds to comparable predicted survival estimates; therefore, this marginal discrepancy is considered clinically negligible and unlikely to confound the primary perioperative outcomes reported in this study.

Additionally, the lack of long-term follow-up data precludes conclusions about mid- or long-term complications as marginal ulcers, biliary reflux, weight loss maintenance or nutritional outcomes.

Future studies with larger cohorts and extended follow-up are needed to validate these findings.

## Conclusions

In conclusion, our findings suggest that r-OAGB and l-OAGB are comparable techniques in terms of operative time, although slightly longer in the robotic group, and postoperative complications. While the differences did not reach strong statistical significance in our cohort, a modest reduction in length of hospital stay, together with lower postoperative pain observed in the robotic group, may contribute to improved short-term outcomes and potentially reduce indirect healthcare costs.

Although robotic surgery is associated with higher direct costs, these may be offset in selected patient populations, particularly those with higher surgical complexity. Future research should focus on high-risk cohorts to better delineate the potential benefits and cost-effectiveness of robotic technology in bariatric surgery and have to evaluate long-term outcomes, including weight loss maintenance, nutritional profiles, and cost-effectiveness analyses, to fully elucidate the role of robotics in the evolving landscape of metabolic surgery.

## Supplementary Information

Below is the link to the electronic supplementary material.

Supplementary file1 (MP4 440128 KB)
